# Maternal effects on offspring growth indicate post-weaning juvenile dependence in chimpanzees (*Pan troglodytes verus*)

**DOI:** 10.1186/s12983-019-0343-8

**Published:** 2020-01-07

**Authors:** Liran Samuni, Patrick Tkaczynski, Tobias Deschner, Therese Löhrrich, Roman M. Wittig, Catherine Crockford

**Affiliations:** 1 Department of Primatology, Max Planck Institute for Evolutionary Anthropology, 04103 Leipzig, Germany; 2 Taï Chimpanzee Project, Centre Suisse de Recherches Scientifiques, Abidjan, Ivory Coast; 3 Department of Human Evolutionary Biology, Harvard University, Cambridge, UK; 4 World Wide Fund for Nature, Dzanga Sangha Protected Areas, Bangui, Central African Republic; 5 Epidemiology of Highly Pathogenic Microorganisms, Robert Koch Institute, Berlin, Germany

**Keywords:** Life history, Orphan, Muscle mass, Hominin evolution, Creatinine, Female dominance

## Abstract

**Background:**

In animals with altricial offspring, most growth occurs after birth and may be optimized by post-natal maternal care. Maternal effects on growth may be influenced by individual characteristics of the mothers, such as social status, individual investment strategies and the length of association with offspring. The prolonged juvenile dependence seen in humans is a distinctive life history adaptation, which may have evolved to facilitate sustained somatic and brain growth.

In chimpanzees, offspring are typically weaned at approximately 4 years old, yet immature individuals continue to associate with their mothers for up to 10 years beyond weaning. Whether this lengthy association or the individual characteristics of mothers influences growth patterns in this species is not clear.

The relationship between urinary creatinine and specific gravity is an established non-invasive measure of muscle mass in humans and chimpanzees. We analysed the urinary creatinine and specific gravity of 1318 urine samples from 70 wild chimpanzees from the Taï Forest, Ivory Coast aged 4 to 15 years.

**Results:**

We showed a clear increase in urinary creatinine levels with age in both males and females, replicating established growth curves in this species and reaffirming this measure as a reliable proxy for lean body mass. Comparing those who experience maternal loss (orphans) with non-orphan chimpanzees, maternal presence beyond weaning age and into late juvenility positively influenced offspring muscle mass throughout ontogeny such that orphans had significantly less muscle mass than age-matched non-orphans. In age-matched offspring with mothers, those with high-ranking mothers had greater muscle mass. Accounting for variation in muscle mass attributable to maternal presence, we found no effect of maternal investment (length of inter birth interval, from own birth to birth of following sibling) on offspring muscle mass.

**Conclusion:**

Chimpanzee mothers have an extended and multi-faceted influence on offspring phenotypes. Our results suggest that maternal investment extends beyond lactation and into early adulthood and has clear benefits to offspring physical development. Therefore, prolonged juvenile dependence, although unique in its form in human societies, may be a trait with deeper evolutionary origins.

## Background

Mammalian life history is characterised by an initial dependence on mothers as the primary source of nutrition [1]. In some species, this is followed by an extended period of association between mother and offspring, during which mothers can vary in their degree of investment, including manipulation of post-natal provisioning [2–4], time allocation to behavioural interactions with their young [5–7], and latency to wean current offspring and seek new mating opportunities [8–13].

Although there is great variation among mammal species in terms of the duration and extent of post-natal maternal effects, human life history is considered distinctive due to the length of juvenile dependence on mothers [14, 15]. This extended mother-offspring association and sustained provisioning in humans is considered adaptive; it facilitates sustained somatic and brain growth, as well as providing extensive social learning opportunities for offspring [14], to the extent that early-life conditions and maternal or care-giver relationships can be key determinants of adult physical and psychological phenotypes in humans [16, 17]. Evidence from other mammalian species suggests that maternal presence beyond weaning positively influences offspring survival [18–21]. With the exception of humans, certain cooperative breeders and obligate carnivores, post-weaning food provisioning of offspring in mammals varies from rare to absent [22–24]. Thus, whether post-weaning maternal presence enhances offspring physical development remains unclear.

Offspring growth trajectories are influenced by characteristics and investment strategies of mothers, such as maternal age, dominance rank, and the latency to wean offspring [25–27]. For instance, high-ranking or more experienced females may have better access to resources compared to younger subordinates [28–32], and greater resource access in high-ranking mothers likely translates into more abundant resources for their offspring. Thus, high-ranking mothers may be able to invest more extensively in offspring compared to subordinates, with likely implications for offspring growth and muscle development. While rank is a known predictor of reproductive success for mothers in many mammal species in terms of infant survival and fertility [33–35], relatively little is known about how rank might predict variation in muscle mass in offspring throughout development. Studies of monkey species [36, 37] and some other large mammals [38] show a positive relationship between maternal rank and offspring growth rates, however, research on this topic in one of our closest living relatives, chimpanzees, is currently lacking.

Chimpanzees are a model species for the study of the evolution of maternal effects on early life history strategies as offspring show regular and prolonged associations with their mothers relative to most primate and mammal species [39, 40], allowing investigation of both post-natal and post-weaning maternal investment and its effects on offspring growth. However, lengthy periods of immaturity in chimpanzees have, until recently, limited the ability for researchers to study these effects with the same level of detail as has been achieved in shorter-lived mammals [41, 42].

In our study, we used two decades of data to investigate the potential effect of maternal presence, dominance rank and investment on variation in muscle mass in pre-adult wild chimpanzees (4–15 years old) in the Taï National Park, Côte d’Ivoire. Previously, Emery Thompson et al. 2012a, demonstrated that the relationship between specific gravity (SG) and creatinine content of urine samples can be used to non-invasively assess muscle mass, generating growth curves that reflect established weight curves for chimpanzees [43]. Creatinine is the by-product of cellular metabolic activity occurring in muscle tissue: creatine and phosphocreatine dehydrate into creatinine at low and constant rates which is subsequently excreted in urine, thus the more muscle tissue an individual has, the greater the quantities of creatine and thus greater the rate of creatinine excretion [44].

Based on those findings, for our study, we predicted muscle mass, as measured by SG-corrected urinary creatinine content, would increase throughout development in immature chimpanzees. As chimpanzee are moderately sexually dimorphic, and males have relatively larger body sizes than females [45, 46], we predicted muscle mass development to be faster in male subjects compared to females. Maternal investment and characteristics are thought to influence offspring phenotype, however, a recent cross-species meta-analysis found that the presence of maternal care had a limited effect on the amount of trait variation attributable to maternal effects within a species [47]. In many mammals, post-weaning maternal care is often short-lived, which has limited efforts to fully explore maternal effects beyond the pre-natal or the immediate, post-natal period ([48–50]; although see [20]). Here, with a sample of wild chimpanzees that were naturally orphaned post-weaning or continued to be mother-raised until adulthood, we have a unique opportunity to examine the impact of maternal presence and investment on offspring growth during this specific life stage. We predicted that the presence of the mother influences offspring growth, such that immature chimpanzees with living mothers at the time of sampling would have higher levels of muscle mass compared to immature chimpanzees that lost their mother (i.e., orphans). Orphan chimpanzees lack maternal provisioning or maternal investment (in terms of food sharing or support during conspecific aggression for example [51–54]), which may affect their ability to allocate resources towards muscle mass development.

Maternal characteristics, such as dominance rank, have been previously associated with fitness gains [38, 55, 56]. However, the mechanisms upon which maternal rank and fitness link have received less attention. Dominance rank in chimpanzees may determine priority of access to valuable resources with potential implications for maternal investment and thus effects on offspring growth. We predicted that increased dominance rank of mothers would translate into higher muscle mass in offspring. Although linear dominance hierarchies exist in females in our study population [57], it is not clear that priority of access to resources for females should also increase in a linear fashion in correlation with increasing rank. We anticipated that the most clear delineation in priority of access to resources would be between the highest-ranking mothers compared with lower-ranking mothers, as has been demonstrated in other populations [58].

Beyond their own characteristics (such as rank), the individual investment strategies of mothers may affect offspring development. In our final analysis, we predicted that females that invest more energy in their offspring’s upbringing would conceive later, reflected by the duration of the inter-birth interval (IBI) between that offspring and the subsequent birth. Thus, we expected that higher maternal investment and hence longer IBIs, would lead to higher levels of muscle mass in offspring [41].

## Methods

### Data collection

Data were collected at the Taï National Park, Côte d’Ivoire (5°45′N, 7°7′W [59]) on three different chimpanzee communities (i.e., North, South and East). Systematic observation effort, including collection of demography data in Taï, started with North group in 1982 ([53]; North group: 1982-present; South group: 1993-present; East group: 2000 – present). Behavioural data included nest to nest focal-follows [60] of individuals of the different social groups on a daily basis by trained local assistants and researchers. In addition to focal follows, observers regularly collected urine samples from young individuals (70 subjects, 4 to 15 years) between February 2000 and July 2018.

Urine sample collection and demography data were used to investigate the effect of maternal presence and investment on offspring lean muscle mass. Weaning in chimpanzees is estimated to occur at around 4–5 years of age [61, 62]. Post-weaning, individuals typically stay in regular association with their mother up until the age of 10 years, around which time they gradually become fully independent: males begin integrating into the dominance hierarchy of the group and females are increasingly likely to disperse from their natal group [53, 63]. Accordingly, we defined maternal loss (i.e., orphans; *n* = 18) as individuals who lost their mother post-weaning and before 10 years of age [54], and investigated the influence of maternal dominance rank (see below) in individuals under 10 years, taking into account the period of mother-offspring association in chimpanzees. In accordance with published weaning age estimates [61, 62], in our study population, the youngest age a post-weaned orphan was sampled in our dataset was at 4.0 years old. Therefore, and in line with previous study [41], we restricted subsequent analyses to samples collected from individuals between the ages of 4 to 15 years. We could determine offspring IBIs (to subsequent births) for 54 individuals, using demography data. We had reliable information of the year and month of birth for all individuals (including the study subjects and their siblings for IBI calculations). In cases where the day of birth was unknown, we assigned the 15th of the respective birth month as the day of birth. The IBI for an individual was calculated as the exact time in years between their birth and the birth of the subsequent offspring of their mother.

### Dominance rank

To determine the dominance relationships between adult females in each community, we used submissive uni-directional pant grunt vocalizations [57] (North: 966 vocalizations, South: 1302 vocalizations, and East: 207 vocalizations), and applied a likelihood-based adaptation of the Elo rating approach [64–66]. Within each social group, we then distinguished maternal ranks in two ways. First, for each offspring, we assigned a continuous Elo score of their mother’s rank (standardized between 0 and 1 in each group) calculated on the date of each offspring urine sample. Second, as rank effects may be non-linear, particularly in terms of resource acquisition in female chimpanzees, we also delineated between females of the highest dominance rank (alpha), and females with a rank other than alpha (subordinate). This is in accordance with other studies of female chimpanzee rank [30, 32, 58, 67], and follows previous findings of higher and more constant body mass in the highest ranking chimpanzee females [58].

### Urine sample collection and creatinine and SG analyses

Urine samples were collected in the field into 2 ml cryo vials from leaf litter using a plastic pipette. Upon arrival in camp, and within 12 h of collection, samples were transferred into liquid nitrogen. Samples were then shipped frozen on dry ice to the Laboratory of Endocrinology at the Max Plank Institute for Evolutionary Anthropology in Leipzig, Germany, where we stored them at − 80 °C until analysis.

Creatinine levels were measured via colorimetric reaction of urine with picric acid. To account for the concentration of urine, we measured SG, which is independent of muscle mass, using a digital refractometer (TEC, Ober-Ramstadt, Germany). Following common practice, we exclude highly diluted urine samples. Thus, we excluded 137 samples with SG < 1.003 [41], and 16 additional samples with creatinine levels with values ≤0.05 mg/ml [68]. We estimate muscle mass of young individuals (4–15 years) by means of creatinine controlling for SG for each urine sample. This is a pre-established validated measure in chimpanzees [41] and humans [44].

### Statistical analysis

To investigate the effects of age, sex, maternal presence, rank and investment on offspring muscle mass (log transformed creatinine mg/ml) we fitted Linear Mixed Models (LMM) [69] with Gaussian error structure and identity link function.

In the first model (‘effects of maternal presence’), we investigated the effect of maternal presence or absence on offspring muscle mass during the development of immature male and female chimpanzees. Our test predictors for this model were the interaction between the age and sex of the individual, as well as whether the individual was an orphan or not at the time of urine sample collection. Furthermore, to account for urinary concentrations, we included SG (minus 1) as a control predictor [41]. As seasonal variation in rainfall, temperature and humidity may influence creatinine levels in Taï [70], we controlled for circannual variation in creatinine levels by converting Julian dates into a circular variable, and including its sine and cosine into the model [70, 71]. We included group membership as another control predictor (i.e., North, South, or East). Furthermore, as we use repeated measures from individuals, offspring with the same mother and days, we included the identity of the subject, its mother, the year (combination of group and year, termed “year id” hereafter) and day (combination of group and date, termed “day id” hereafter) as random effects. By this we account for non-independent sampling of certain subjects, their mothers, days or years disproportionally affecting urinary creatinine levels, and thus, avoid pseudo-replication [69]. In order to keep type I error rate at the nominal 5% and to account for potential non-uniform variation of our predictor variables within the random effects [72, 73], we included a maximal random slope structure, incorporating random slopes for the predictors with appropriate variation within the particular random effects. This resulted in random slopes for age, SG, and sine and cosine of date within subject, mother and year id. Our dataset for the ‘effects of maternal presence’ model included 1318 urine samples of 70 individuals (a mean + SD of 18.83 + 19.15 samples per subject) and 41 mothers.

In the second LMM (‘maternal rank effects’), we investigated the effect of maternal dominance rank on the development of offspring muscle mass. We used submissive uni-directional pant grunt vocalizations [57] to calculate the dominance rank of mothers. Maternal dominance rank may affect offspring muscle mass through priority of access to high quality food sources of high-ranking mothers. Thus, in this model we included samples of offspring with known maternal rank and aged between 4 and 10 years to reflect the estimated period that young, weaned chimpanzees regularly associate with their mothers [53]. The ‘maternal rank effects’ model included all the predictors from the ‘effects of maternal presence’ model with the exception of the predictor ‘orphan (yes/no)’, as this model only included samples from individuals whose mother was alive. Our test predictors for this model were the dominance rank of the mother for each subject’s sample as both a continuous (linear) and a categorical term (alpha vs. subordinate). To evaluate reliably the effect of maternal rank on muscle mass, independently of maternal age, we included an additional control predictor of the age of the mother into the analysis (this showed no collinearity with maternal dominance rank: vif < 1.6). As per the ‘effects of maternal presence’ model we included the random effects of subject, mother, year id and day id, as well as random slopes for the age of the subject, age and rank of the mother, SG and seasonality variables within subject, mother and year identity. Our dataset for the ‘maternal rank effects’ model included 414 urine samples of 48 subjects (a mean + SD of 8.62 *+* 8.51 samples per subject) and 29 mothers.

In our final analyses, to evaluate the effect of maternal investment on offspring development, we extracted the variance of the intercept of the random effect of individual identity (‘best linear unbiased predictors’ [74]) from the ‘effects of maternal presence’ model (this includes both orphans and non-orphans between 4 and 15 years of age). We only included subjects with known IBI and at least 2 urine samples (*n* = 45 subjects; a mean + SD of 19.20 + 20.68 samples per subject). We then fit a linear regression for each sex of these variance estimates against IBIs, our proxy measure for maternal investment [8, 41]. By taking this approach, we were able to investigate the independent effect of IBI on muscle mass while accounting for the effects of age, sex and maternal presence on muscle mass. This approach was preferred over including IBI in either the ‘effects of maternal presence’ or ‘maternal rank effects’ models as this would have limited the number of individuals that could be included given either IBI is unknown or absent (they are the sole dependent offspring of their mother during the sampling period) for many individuals included in these models. The best unbiased linear predictors were extracted from the ‘effects of maternal presence’ model rather that the ‘maternal rank effects’ model as the former contained a larger number of individuals and samples as well as greater variance attributable to the random effect of individual identity than observed in the latter model.

For all statistical analyses, we used R (version 3.5.3 [75]) to process the data and fit the models. Prior to fitting the models, we checked the distribution of the response and all predictors. As a result, we log transformed creatinine levels to achieve a more symmetrical distribution. In addition, we z-transformed the covariates of IBI, SG, age of subject, and age and dominance rank of mother [76]. We verified the assumptions of normally distributed and homogeneous residuals by visual inspection of qq-plots and residuals plotted against fitted values. These evaluations did not reveal obvious deviations from model assumptions. We used the function *vif* of the R package ‘car’ [77] applied to a standard linear model lacking the random effects to derive Variance Inflation Factors (VIF), which did not reveal collinearity problems (largest VIF: ‘development model’ = 1.20**; ‘**maternal effect model’ = 1.89 [78]).

We used the function *lmer* of the R package ‘lme4’ [79] to fit both models. We compared the fit of both full models with those of a respective null model lacking only the test predictors (thus the null model contains all control predictors, random effects and slopes), using a likelihood ratio test [80]. We obtained individual *p*-values for the fixed effects using the *drop1* function in R, by systematically dropping each fixed effect from the model one at a time [72], and comparing the respective reduced model lacking the individual fixed effects with the full model. For both models, we assessed model stability by excluding the levels of the random effects (identities of subject, mother, day id, year id) one at a time and comparing the estimates derived for these subsets with those derived for the full data set. Stability assessment revealed no influential subjects, day or year id to exist. To obtain confidence intervals we conducted parametric bootstraps using the function *bootMer* of the package ‘lme4’. Finally, we evaluated the effect sizes (R^2^) using the function *r.squaredGLMM* of the R package ‘MuMIn’ [81]. We report the variance explained by the fixed effects (marginal-R^2^_m_), and the fixed and random effects (conditional-R^2^_c_) [82].

## Results

**Fig. 1:**
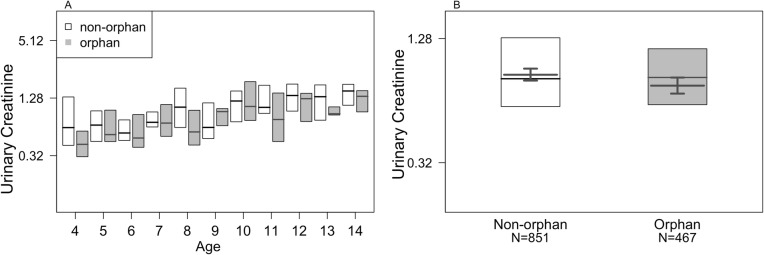
The effect of maternal presence on creatinine levels (mg/ml) of offspring between the ages of 4–15 years (*n* = 1318 samples). The figure panels depict the data by **a** age and orphan status or B) orphan status alone. In both panels the thin black horizontal lines represent medians and the white (non-orphan) and grey (orphan) boxes represent quartiles. In **b**, the thick black lines represent the fitted model values and the black error bars its 95% confidence intervals

The ‘effects of maternal presence’ model examining the potential effects of sex, age and maternal presence on urinary creatinine levels of weaned individuals (while accounting for the concentration of the urine through SG) showed significant results (LMM: full-null model comparison - likelihood ratio test: χ2 = 59.345, df = 4, *P* <  0.001). We found that maternal loss in weaned individuals had a significant negative effect on offspring’s muscle mass, such that orphans had lower muscle mass compared with non-orphans (*P* = 0.012; Fig. 1; Table 1).

**Table 1:** ‘Effects of maternal presence’ model results**:** effects of maternal presence on creatinine levels (log-transformed)

Term	Reference category	Estimate	SE	95% CI	Chisq^*^	P
Intercept		−0.281	0.071	−0.430, − 0.144	**–**	**–**
*Test predictors*						
Age ^a^		0.134	0.044	0.037, 0.227	**–**	**–**
Sex (Male)	Female	0.100	0.047	0.001, 0.191	**–**	**–**
Age ^a^: Sex (Male)	Female	**0.162**	**0.047**	**0.092, 0.272**	**10.510**	**0.001**
orphan (Yes)	No	**−0.122**	**0.046**	**− 0.220, − 0.027**	**6.376**	**0.012**
*Control predictors*						
SG^b^		**0.690**	**0.024**	**0.641, 0.740**	**131.566**	**< 0.001**
North group	East group	−0.104	0.081	−0.263, 0.060	4.129	0.127
South group		0.047	0.073	−0.110, 0.195		
Sine Julian date		**0.053**	**0.030**	**−0.006, 0.117**	**27.019**	**< 0.001**
Cosine Julian date		**0.186**	**0.031**	**0.120, 0.250**		

Statistically significant effects (*P* ≤ 0.05) appear in bold and coded level of factors in parenthesis

^a-b^z-transformed, mean ± SD of the original variable: ^a^ 9.61 ± 3.26 years, ^b^1.02 ± 0.01

^*^df = 1 except for Group, where df = 2

**Fig. 2:**
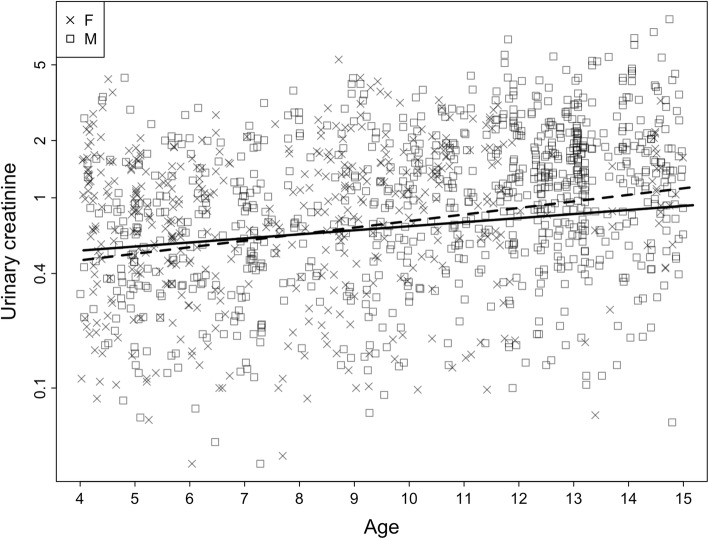
Effect of the two-way interaction between the age and the sex of the subject on urinary creatinine levels (mg/ml; *n* = 1318 samples). Shown are the creatinine values for **a** males (squares; 39 subjects) and **b** females (crosses; 31 subjects). The solid and dashed lines represent the model line for females and males, respectively

We found a significant two-way interaction between age and sex on creatinine levels (P <  0.001; Fig. 2; Table 1). Age had a positive association with creatinine concentrations in female subjects (Estimate ± SE: 0.134 ± 0.044, 95% confidence intervals: 0.037–0.227) and we observed a more pronounced effect of age on muscle mass in males compared with females (Estimate ± SE: 0.162 ± 0.047, 95% confidence intervals: 0.092–0.272). This is despite females typically having comparable muscle mass with males up to the ages of approximately 9 years old (Fig. 2). As expected, we found a significant effect of SG (*P* < 0.001) and seasonal variation in creatinine levels (P < 0.001). The identity of the social group had no effect on creatinine levels of young individuals. The overall variance explained by the fixed effects was R^2^_m_ = 0.66 and by the random and fixed effects was R^2^_c_ = 0.84.

**Fig. 3:**
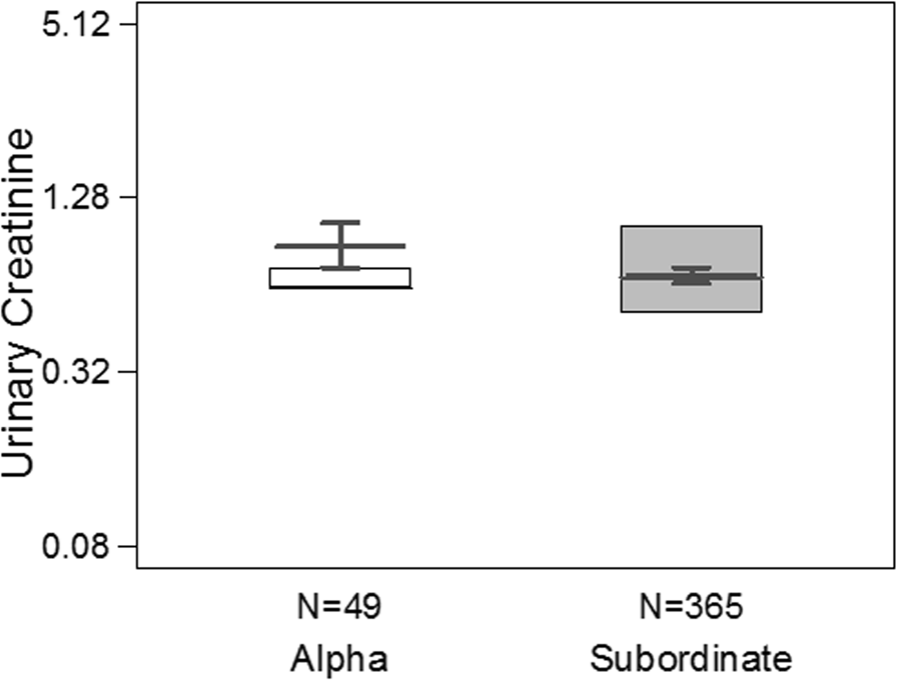
Effect of mothers’ dominance rank (i.e., alpha vs. subordinate) on urinary creatinine levels of offspring between the ages 4–10 years (*n* = 414 samples). Shown are the medians (thin horizontal lines), quartiles (boxes) and the fitted model (thick grey lines) and its 95% confidence intervals (grey error bars) as obtained from the ‘maternal rank effects’ LMM

The ‘maternal rank effects’ model (full-null model comparison likelihood ratio test: χ2 = 7.809, df = 2, *P* = 0.020; Table 2) showed maternal rank influenced muscle mass development in weaned offspring. Specifically, the categorical term of dominance rank had a significant positive effect on offspring muscle mass (*P* = 0.006; Fig. 3), while we found a weak trend for the effect of the continuous term of dominance rank (*P* = 0.093; Additional file 1: Figure. S1) on offspring muscle mass. Specifically, offspring of subordinate mothers had lower muscle mass in comparison to offspring of alpha females. Furthermore, we found a significant effect of SG (*P* < 0.001), and seasonal variation (*P* = 0.005) on urinary creatinine levels. The age of the mother, the two-way interaction between age and sex, and group identity had no significant effect on creatinine levels. The overall variance explained by the fixed effects of the model was R^2^_m_ = 0.71 and by the random and fixed effects was R^2^_c_ = 0.90.

**Table 2:** ‘Maternal rank effects’ model results: The effect of mother’s dominance rank on creatinine levels (log-transformed)

Term	Reference category	Estimate	SE	95% CI	Chisq^*^	P
Intercept		−0.090	0.105	−0.298, 0.135	–	–
*Test predictors*						
Categorical mother’s rank (Subordinate)	Alpha	**−0.292**	**0.103**	**−0.491, − 0.090**	**7.632**	**0.006**
Continuous mother’s rank		−0.054	0.031	−0.117, 0.011	2.828	0.093
*Control predictors*						
Age ^a^		0.147	0.036	0.068, 0.220	**–**	**–**
Sex (Male)	Female	−0.180	0.059	− 0.298, − 0.059	**–**	**–**
Age ^a^: sex (Male)	Female	−0.061	0.050	−0.162, 0.038	**1.407**	**0.236**
SG^b^		0.797	0.057	0.683, 0.917	69.257	< 0.001
Mother’s age^c^		0.061	0.035	−0.012, 0.132	2.671	0.102
East group	North group	0.151	0.078	0.007, 0.308	3.698	0.157
South group		0.080	0.069	−0.056, 0.220		
Sine Julian date		**−0.034**	**0.055**	**−0.142, 0.084**	**10.274**	**0.005**
Cosine Julian date		**0.144**	**0.046**	**0.045, 0.237**		

Statistically significant results (P ≤ 0.05) appear in bold and coded level of factors in parenthesis

^a^z-transformed, mean ± SD of the original variables: ^a^6.62 ± 1.82 years, ^b^1.02 ± 0.01, ^c^30.52 ± 8.30 years

^*^df = 1 except for Group, where df = 2

**Fig. 4:**
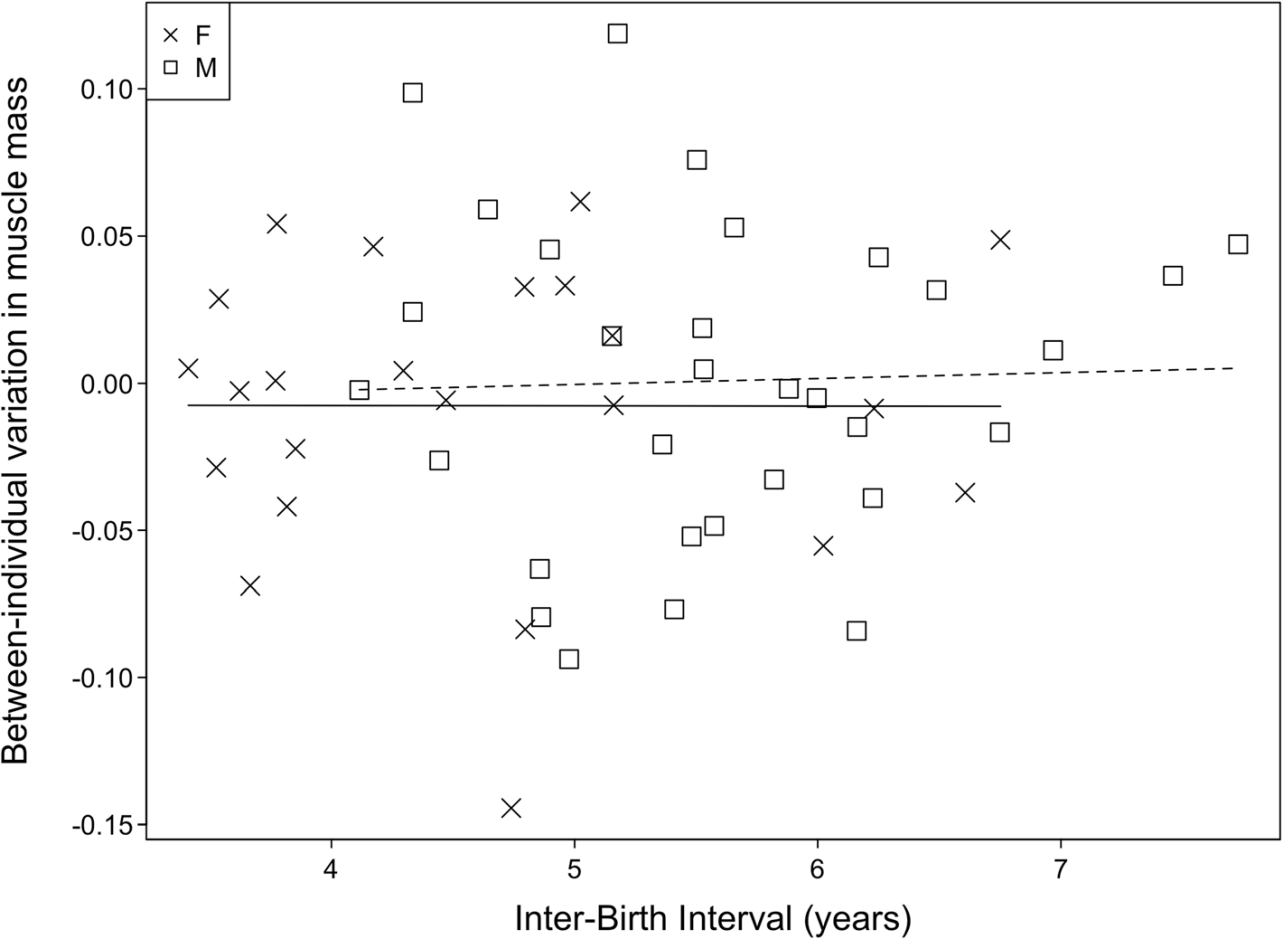
Association between inter-birth interval (next offspring) and estimated between-individual variance in creatinine levels in males (squares, *n* = 31) and females (crosses, *n* = 23). Points indicate variance of the random effect of subject as obtained from the ‘effects of maternal presence’ model (taking into account subjects’ age and sex, the urine sample’s specific gravity, maternal loss, group identity, and seasonal variation). The solid or dashed lines represent the regression of relative muscle mass on inter-birth interval in females and males, respectively

We investigated the effect of maternal investment strategies (IBI) on male (*n* = 31) and female (*n* = 23) offspring muscle mass using best linear unbiased predictors of the random effect of individual identity (as obtained from the ‘effects of maternal presence’ model). This estimated individuals’ creatinine levels relative to all other individuals while accounting for variation attributable to age, sex, orphan status and control predictors. IBI was significantly (independent-samples t test: 95% confidence intervals 0.453–1.523; t_*43.82*_ = 3.721; *P* < 0.001) longer in males (mean ± SD: 5.6 ± 0.9 years) in comparison with females (4.6 ± 1.0 years). After accounting for maternal presence, sex, and age of the offspring we found no significant effect of IBI on between-individual variation in muscle mass in female (*P* = 0.968) or male (*P* = 0.934) offspring (Fig. 4).

## Discussion

Maternal investment can have sustained effects on whether and how individuals can allocate resources to the development of muscle mass. In humans, unlike in most other animals, maternal investment continues years after weaning, into and during puberty [83]. In our study, we demonstrate that these long-term maternal effects, rarely shown outside of humans [20], are also evident in one of our closest living relatives, the chimpanzee, with implications for our understanding of life history adaptations in hominins [84].

We found that young male and female chimpanzees demonstrated a pattern of increasing muscle mass with increasing age. This result both confirms our primary prediction, replicates a previous study [41], and provides further biological validation for the use of urinary creatinine and SG concentration as a method for non-invasive monitoring of muscle growth in wild chimpanzees and potentially other animals [43]. Males generally had steeper growth patterns than females and by approximately 9 years of age, males on average had more muscle mass than females. Adult chimpanzees are moderately sexually dimorphic in terms of body size, with males typically larger and heavier than females [45, 46]. The ontogenetic pattern of sexual dimorphism in our study is in line with former anatomical and veterinarian studies conducted with captive chimpanzees which show steeper growth curves for males [46, 85–87].

Weaning in chimpanzees is estimated to occur at around 4–5 years old [61, 62, 88]. In most mammals, with the exception of humans, it is assumed that weaned individuals face an immediate pressure to become rapidly independent and receive limited provisioning from their mothers [56]. Food sharing between chimpanzee mothers and weaned offspring is irregular, with continuous provisioning ceasing during the weaning phase [62]. Nevertheless, in our study, we demonstrate that the loss of a mother has long-lasting implications for growth in wild chimpanzees, even when occurring after weaning age, providing evidence for an indirect but prolonged juvenile dependency in chimpanzees. Unlike in many human societies, alloparenting in wild chimpanzees is rare [89]. However, chimpanzees sometimes ‘adopt’ orphans, manifested in carrying, sharing nests and food and supporting orphans during aggressive events [51, 54]. Orphans that are adopted may have better survival outcomes than those that are not [54], but whether adoption can partially compensate the physical costs of maternal loss remains to be tested using a larger dataset (7 of the 18 orphans included in our study were adopted [51]).

Nonetheless, our results show that the loss of a mother significantly limits muscle growth and that the maternal effects on physical growth persist years after weaning, throughout the juvenile period and into puberty in chimpanzees. There are multiple, nonexclusive mechanisms which could lead to reduced muscle mass in orphaned chimpanzees. First, without maternal (or allo-parental) agonistic support, orphans may have less access to valuable food resources such as meat and nuts, which might provide essential micronutrients required for the development of muscle mass [90, 91]. Second, orphans may need to allocate resources away from growth and towards survival through energetically costly behaviours such as independent travel and foraging, without the aid of maternal buffering in competitive interactions. These behavioural transitions are expected to be associated with increased physiological and psychological stress through elevated levels of circulating glucocorticoids: these hormones stimulate metabolic processes to allow the rapid redirection of behaviour to address stressors and return normal homeostatic function [92, 93]. However, glucocorticoids also have an inhibitory effect on the immune system and growth [93–95]. Thus, maternal loss may lead to increased exposure to physical and psychological stressors for orphans, coupled with reduced access to high-value food items, leading to sustained and elevated physiological stress and thus suppression of growth and muscle development [95]. Concurrent to this, the potential shift in resource prioritization to survival over growth could see energetic intake utilized for the development of adipose tissue and fat reserves rather than increased musculature, such as occurs in ageing animals or those moving between reproductive and winter (challenging) seasons [96, 97]. Whilst the pattern that a mother’s death results in reduced post-weaning growth, behavioural and hormonal data are required to understand what continued maternal presence provides and what limits muscle growth in orphaned compared to non-orphaned chimpanzees, particularly in those individuals orphaned post-weaning.

The ‘effects of maternal rank’ model confirmed our prediction that individual characteristics of chimpanzee mothers, i.e. rank, are associated with patterns of muscle growth in their offspring. Specifically, alpha females (controlling for the effect of mother’s age) had offspring with higher muscle mass compared to the offspring of low-ranking mothers. When using a continuous rank variable, we found only a trend for increasing maternal rank to be associated with offspring muscle mass. While there is indirect evidence from other chimpanzee populations that high-ranking female chimpanzees occupy higher quality locations within a group’s territory at the expense of subordinates [32], Taï chimpanzees are considerably more cohesive than other populations and females do not occupy particular areas within a group’s territory [53, 98, 99]. This relatively high cohesiveness could be driven by a combination of both higher food availability and greater predation pressure than at other sites [100]. High food availability would potentially enable females to socialise in larger parties without dramatically increasing food competition, thus limiting the effect of rank on resource acquisition in general.

Taï chimpanzees also supplement their diet with a number of high-value, limited, food resources, namely meat and nuts [53], which are either monopolisable in and of themselves (meat) or the tools for their extraction may be monopolisable [101]. Thus, as these high quality resources are limited in space and time, it is likely that access to such resources is not proportionally dependent on dominance rank but rather more prominent and predictable in the alpha females compared with subordinates. This is supported by evidence from another chimpanzee population showing greater body mass in the highest ranking female chimpanzees [58]. Indeed, during periods of relative food scarcity in Taï, high-ranking females remain gregarious, while low-ranking female become temporarily more peripheral [67]. This suggests that high-ranking females are able to acquire sufficient resources regardless of circumstance and thus do not need to change foraging or social strategies to the same extent as low-ranking females. However, whether the apparent benefits of high rank directly translate into offspring receiving greater access or amounts of high-value food items requires detailed behavioural data from the offspring themselves.

With the current dataset we cannot exclude the possibility that the relationship between high rank and offspring muscle mass is genetic, i.e. females with a genetic predisposition for greater muscle mass tend to achieve high ranks, and it is this heritable predisposition for muscle mass that is leading to higher muscle mass in their offspring relative to the offspring of low-ranking individuals. However, heritability estimates for muscle mass in humans and other large animals tend to be low [102, 103], and chimpanzee females typically queue rather than compete for dominance rank [65]. Therefore, genetic effects are an unlikely mechanism explaining the patterns observed in our study. Overall, our results show that potential rank-based advantage experienced by females in Taï may also translate into increased muscle mass for offspring.

In our final analysis, we were unable to replicate the results of Emery Thompson et al. (2016), who found a significant relationship between the muscle mass of an individual and the IBI between its own and its subsequent sibling’s birth. Lactation is energetically costly, and subsequent conception in chimpanzees is predicted by the energy balance of mothers [88]. Therefore, longer IBIs may not only represent an increase in the time mothers invest in their current offspring, but also increased energy invested. Chimpanzee mothers typically have longer IBIs for male compared with female offspring, and in our study, the IBI for males was on average 25% longer than that of females. As conception is tightly linked with the energetic balance of mothers, longer IBIs of males suggest that mother’s invest more energy in sons than in daughters. However, given that there was no general effect of IBI on muscle mass, we conclude that the extra maternal investment in sons is required to keep similar levels of muscle mass to that gained by daughters following shorter IBI. A previous study of the Taï population identified that dominant females invest more in sons in terms of IBI length, which, rather than being a consequence of sex-dependent costs of investment, was attributed to local resource competition and the benefit of investing in the philopatric sex over the dispersing sex [8]. In our study, males have steeper growth curves than females, suggesting more rapid accumulation of lean muscle mass, which may require greater and more sustained energetic investment from mothers. This pattern is in keeping with evolutionary theories of parental investment, in which there are greater inclusive fitness benefits for mothers to invest heavily when able, into physically competitive (larger muscle mass) and thus potentially reproductively successful sons [27]. However, the results of our analysis suggest that when maternal presence is accounted for in the post-weaning period, the influence of IBI on muscle mass is diminished. Therefore, the post-weaning association between mother and offspring is perhaps the key determinant of muscle mass phenotype in this population of wild chimpanzees.

## Conclusion

Our study highlights the importance of maternal presence, characteristics and investment on the physical development of offspring in chimpanzees. We demonstrate that the importance of maternal presence to offspring development lasts years beyond weaning and that recovery from maternal loss in terms of muscle mass development is negligible during the juvenile phase (although we cannot rule out that orphaned individuals may recover “normal” levels of muscle mass following the period of growth in late-stage puberty and early adulthood). Prolonged juvenile dependence is considered a distinctive characteristic of human societies [14], thought to increase survival during juvenility and early adulthood to support long life spans. Although it remains untested whether maternal presence and investment increase survival of juvenile chimpanzees in our population, our results emphasize that they have a noticeable effect on offspring via the accruement of lean muscle mass, which in turn may translate into future fitness benefits. Thus, although juvenile chimpanzees rely less on adult provisioning (i.e., food sharing) in order to meet their daily energetic needs when compared with humans [14], we suggest that their food consumption is nonetheless indirectly dependent on maternal care. This may occur through mechanisms whereby the presence of mothers buffer offspring against competition and increase offspring access to valuable food resources, which our results suggest may be particularly evident for offspring of high-ranking mothers. Taken together, prolonged juvenile dependence, although unique in its form in human societies, may be a trait with deeper evolutionary origins than previously presumed. Discerning how post-natal maternal behavioural strategies influence growth trajectories in wild chimpanzees is a vital step in understanding how this species maximises the lengthy juvenile phase it shares with few other species.

## Supplementary information

**Additional file 1. Figure S1.** Effect of dominance rank (continuous, 1 being the alpha) on urinary creatinine levels of offspring between the ages 4-10 years (n = 414 samples). Shown are the urinary creatinine levels (larger point areas denote a larger number of samples) and the fitted model (dashed lines) as obtained from the ‘maternal rank effects’ LMM.

## Data Availability

The datasets used and/or analysed during the current study are available from the corresponding authors on reasonable request.

## Abbreviations

IBI: Inter-birth interval
LMM: Linear mixed model
SG: Specific gravity
VIF: Variance inflation factor
