# Supplementary figures and images for: Maternal effects on offspring growth indicate post-weaning juvenile dependence in chimpanzees (Pan troglodytes verus)
Source: Front Zool. 2020 Jan 7;17:1. doi: 10.1186/s12983-019-0343-8 (PMC6945487; doi:10.1186/s12983-019-0343-8)

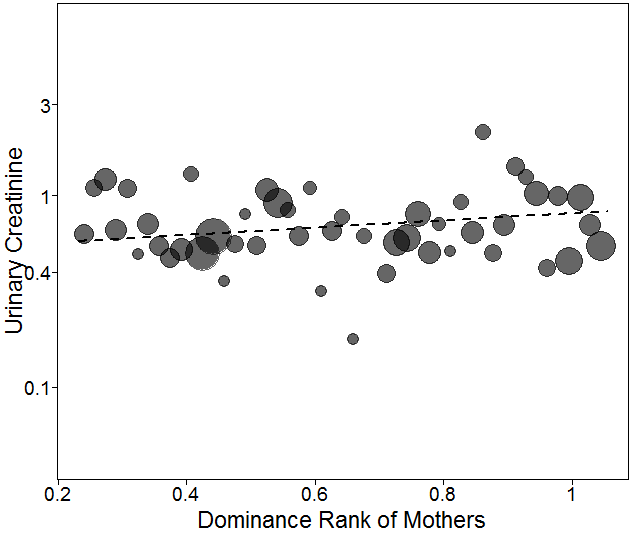

Supplement: Supplementary file 1 — Additional file 1. Figure S1. Effect of dominance rank (continuous, 1 being the alpha) on urinary creatinine levels of offspring between the ages 4-10 years (n = 414 samples). Shown are the urinary creatinine levels (larger point areas denote a larger number of samples) and the fitted model (dashed lines) as obtained from the ‘maternal rank effects’ LMM. [file 12983_2019_343_MOESM1_ESM.tiff]
